# Rapid removal of ammonium from domestic wastewater using polymer hydrogels

**DOI:** 10.1038/s41598-018-21204-4

**Published:** 2018-02-13

**Authors:** Heidy Cruz, Paul Luckman, Thomas Seviour, Willy Verstraete, Bronwyn Laycock, Ilje Pikaar

**Affiliations:** 1 School of Civil Engineering, The University of Queensland, QLD, 4072 Australia; 2 School of Chemical Engineering, The University of Queensland, QLD, 4072 Australia; 3 Singapore Centre for Environmental Life Sciences Engineering, Nanyang Technological University, 637551 Singapore, Singapore; 4 Center for Microbial Ecology and Technology (CMET), Ghent University, Coupure Links 653, 9000 Gent, Belgium; 5 Advanced Water Management Centre (AWMC), The University of Queensland, QLD, 4072 Australia

## Abstract

To date, technologies to recover ammonium from domestic wastewater from the mainstream have not found widespread application. This is largely due to the low ammonium concentrations in these wastewater streams. This paper reports on the use of polymer hydrogels for rapid sorption of ammonium from domestic wastewater coupled with efficient regeneration by mild acid washing. The sorption capacity of the hydrogel was 8.8–32.2 mg NH_4_–N/g, which corresponds to removal efficiencies ranging from 68% to 80% NH_4_–N, increasing proportionally with the initial ammonium concentration. It was, however, unaffected by changes in pH, as the sorption capacity remained constant from pH 5.0–8.0. Importantly, effective regeneration of the hydrogels under mildly acidic conditions (i.e. pH 4.0) was demonstrated with minimal loss in sorption performance following multiple sorption/desorption cycles. Overall, this study highlights the potential of low-cost polymer hydrogels for achieving mainstream ammonium recovery from domestic wastewater.

## Introduction

For the past century, the conventional activated sludge process has been the preferred approach to treating domestic wastewater worldwide^1^, in which ammonium is dissipated to atmospheric nitrogen by sequential oxidation and reduction through a nitrate intermediate (i.e. nitrification and denitrification). Recent innovative technologies have been introduced to lower the energy demand of this process, through short-circuiting the nitrification-denitrification pathway (e.g. anaerobic ammonium-oxidizing bacteria)^2,3^ or simultaneous nitrification-denitrification^4^. Nonetheless, the underlying principle of destroying the energy equivalent present in the reactive nitrogen has remained unchanged^5^.

In addition to the energy-intensive dissipation of nitrogen into unfixed elemental form (N_2_), which contradicts costly industrial efforts to achieve the opposite (i.e. Haber process), such biological nitrogen transformation processes are prone to producing nitrous oxide (N_2_O)^6–8^, a potent greenhouse gas with a 300-fold stronger greenhouse effect than CO_2_^9^. Recent studies have shown that N_2_O emissions can in fact contribute up to ∼80% of the carbon and environmental footprint of WWTPs^6,10–12^. N_2_O formation can be avoided completely by recovering the ammonium from the wastewater rather than converting it biologically to N_2_. Furthermore, nitrogen recovery was found recently to have the lowest environmental footprint among the different treatment schemes (i.e. mainstream anammox, nitrogen recovery and activated sludge)^13^. Hence, a key priority for water utilities in reducing the environmental footprint of their WWTPs should be to directly recover the ammonium from domestic wastewaters rather than transform it, irrespective of innovations in N-biotransformation efficiencies.

To the author’s best knowledge, however, there are no cost-effective methods available to achieve this. To be cost-effective, most of the existing methods such as air stripping, electrodialysis, struvite precipitation and membrane technologies like reverse osmosis, that can be used to recover ammonium, require concentrations above >1000 mg N/L, which is well above the ammonium concentration in domestic wastewater (40–60 mg/L of NH_4_–N)^14^. Ammonium recovery from domestic wastewater using these current technologies is economically prohibitive, with limited practical feasibility when applied to diluted wastewater streams^15^. To overcome this challenge, there is a need for low-cost alternatives for recovering ammonium from domestic wastewater. Adsorption and crystallization are two possible solutions for ammonium recovery in wastewater applications. Crystallization is considered as a less suitable option for mainstream ammonia recovery due to the low ammonia concentrations involved. A sustainable adsorption process, on the other hand, would require a substratum with high binding affinity for ammonia that can be regenerated following exposure to wastewater. The feasibility of ammonium adsorption using zeolites has been explored for ammonium recovery applications since 1970’s^16,17^. However, even as well-established adsorbents with a high affinity for NH_4_^+^, zeolites still have not found widespread implementation in domestic wastewater treatment. The latter is, to a large extent, related to the occurrence of biofouling, inorganic scaling and high chemical requirements in order to achieve sufficient regeneration of the zeolites^18^.

Polymer hydrogels, three-dimensional polymeric networks that can absorb large amounts of water, are an attractive option for overcoming the limitations of zeolites. Over the last sixty years, the properties and applications of hydrogels have significantly expanded from simple cross-linked networks commonly used for simple water absorbents, hygiene products and wound dressings to complex polymer architectures for tissue engineering scaffolds and drug delivery systems^19^. Hydrogel chemistry can be tailored to sorb specific chemical species such as heavy metals^20–22^ phosphates^23,24^, dyes^25^, and ammonium^26,27^. Furthermore, the intensity of binding interaction between hydrogel and target species can be tailored to respond to stimuli such as pH, salinity and temperature (35–80 °C)^19,28–30^. Unlike with zeolites and ion-exchange resins which bind target molecules by displacement of ions of the same charge that are initially in the ion exchange substrate, target molecules can bind with the hydrogels based on complexation, covalent bonding, electrostatic interactions or even physical adhesion. Thus, the release of target species can be easily induced following sorption, allowing for material regeneration without the need for large amounts of chemicals. In fact, a mild washing method to regenerate polymer sorbents was successfully demonstrated very recently^31^. In the context of ammonium recovery, the versatility of hydrogels in terms of sorption characteristics, selectivity, stimulus response, molecular architecture and overall physical characteristics can potentially overcome the limitations of other existing sorbents. To the best of our knowledge, no structured information has been reported so far on such applications of hydrogels in the context of ammonium recovery from wastewater.

We therefore propose hydrogels as an effective and low-cost sorbent for mainstream ammonium recovery from domestic wastewater. In this paper, short-term experiments were performed to evaluate the concept using a commercially available hydrogel to assess ammonium removal efficacies from domestic wastewater as well as their amenability to reuse and regeneration through multiple sorption-desorption cycles. The impact of the wastewater conditions in terms of pH and initial ammonium concentration on the process efficiency was also evaluated.

## Results

### Proof of concept testing

**Figure 1:**
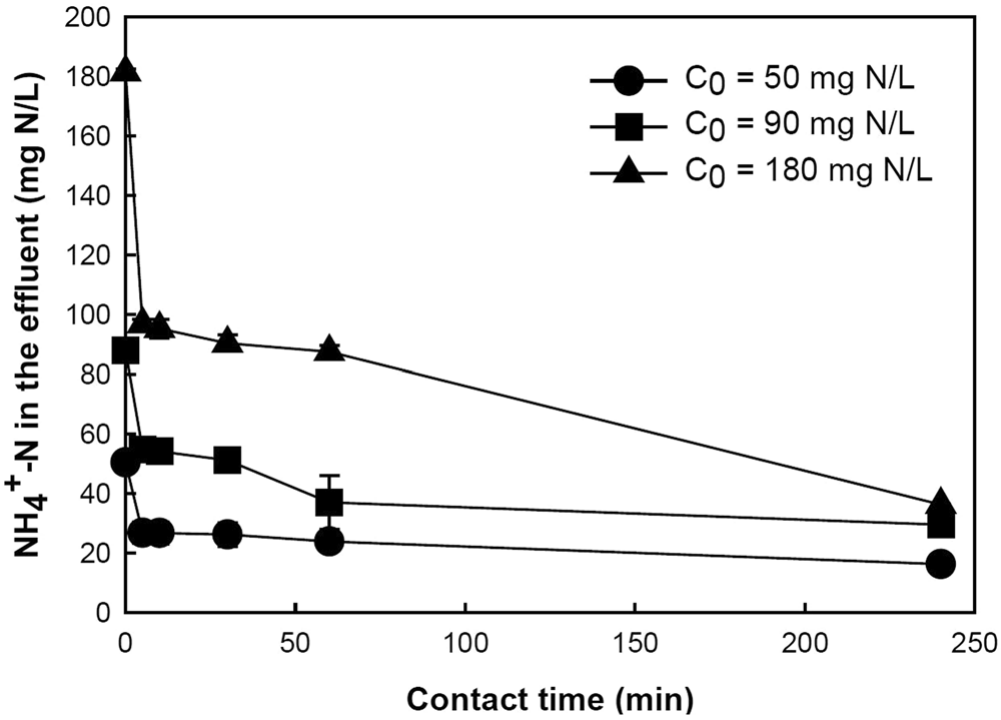
NH_4_-N concentration in the wastewater as a function of time after the addition of PAA hydrogels. The NH_4_-N concentration of the raw wastewater used for this experiment was 50 mg/L. Ammonium chloride was added to increase the NH_4_–N of the wastewater to 90 mg/L and 180 mg/L respectively. Sorption conditions: Contact time = 4 h, T = 23 °C, pH = 7.1. Error bars show the standard deviation from triplicate tests.

The effectiveness of PAA hydrogels in sorbing NH_4_–N from wastewater was initially investigated using raw wastewater. Figure 1 shows that the NH_4_–N concentration decreased with time upon the addition of PAA hydrogels, with the most significant decrease occurring during the first 5 minutes of reaction time. From an initial concentration of 50 ± 0.7 mg NH_4_–N/L, the NH_4_–N concentration was reduced to 16.3 ± 0.3 mg NH_4_–N/L after 4 hours. This corresponds to an average ammonium removal efficiency and sorption capacity of 68 ± 0.7% and 8.8 ± 0.2 mg NH_4_–N/g hydrogel, respectively.

### Effect of initial NH_4_–N concentration and pH

Figure 1 also shows the ammonium uptake profile of PAA hydrogels from domestic wastewater with different NH_4_–N concentrations. The largest reduction in NH_4_–N concentrations occurred within 5 minutes upon the addition of PAA hydrogel, independent of the starting concentration. For higher initial NH_4_–N concentrations (i.e. 90 and 180 mg N/L), the effluent concentrations dropped to 29.5 ± 1.0 and 36.3 ± 0.2 mg N/L, corresponding to NH_4_–N removal efficiencies of 66.4 ± 1.1% and 80 ± 0.01% respectively. Higher sorption capacities were achieved in proportion to the increase in NH_4_–N concentrations, with sorption capacity values of 8.8 ± 0.2, 15.1 ± 0.2 and 32.2 ± 0.2 mg/g for effluents having initial concentrations of 50, 90 and 180 mg N/L respectively. In addition to ammonium, other cations present in wastewater, including magnesium, calcium and potassium, were effectively removed (see Supplementary Table S1). After 4 hours of contact time, the concentrations of Ca^2+^, Mg^2+^ and K^+^ in the wastewater were lowered by 93.6 ± 0.3%, 94.0 ± 0.3% and 70.4 ± 3.0% respectively. By contrast, phosphate anions were not removed at all.

Moreover, the initial pH conditions did not have a significant effect on the sorption capacity of PAA hydrogels (see Supplementary Fig. S2). Within the pH range of 5.0–8.0, the highest sorption capacity observed was 5.8 ± 0.1 mg/g at pH 7.0, and the lowest was 5.1 ± 0.1 mg/g at pH 5.0.

### Regeneration and reusability

**Figure 2:**
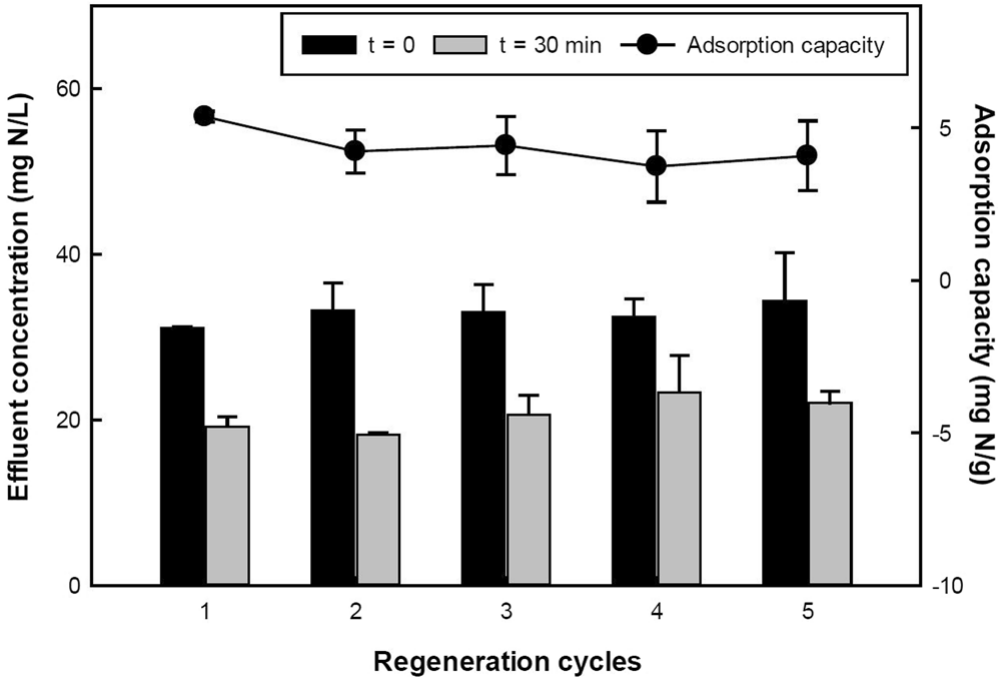
NH_4_–N concentrations before and after sorption, and sorption capacity of the PAA hydrogels as a function of sorption-desorption cycles. Sorption conditions: C_0_ = 33 mg N/L, Contact time = 30 min, T = 23 °C, pH = 7.0. Error bars show the standard deviation from triplicate tests.

Five consecutive sorption-desorption cycles were performed with the regenerated PAA hydrogel exhibiting only a slight decrease in its performance (Fig. 2). The hydrogel was able to sorb an average of 4.3 ± 0.5 mg/g throughout five cycles. Overall, recovery efficiencies of 76 ± 0.8% (first cycle), 76 ± 2.0% (second), 104 ± 0.3% (third) and 109 ± 1.9% (fourth) and 87 ± 6.4% (fifth) were obtained. Higher recovery efficiencies during the third and fourth cycle are likely due to NH_4_^+^ ions that were not desorbed from the hydrogels during the earlier cycles.

## Discussion

We have demonstrated the feasibility of polymer hydrogels as efficient sorbents for mainstream ammonium recovery from domestic wastewater by showing its operational principle. PAA hydrogels primarily contain carboxylic acid groups (–COOH) as functional groups in the polymeric hydrogel network. When immersed in raw wastewater at pH conditions above its pKa (pKa = 4.7), the –COOH groups in the hydrogel become fully ionized to negatively charged –COO^−^ groups which attract positively charged NH_4_^+^ ions present in wastewater through electrostatic interactions which is found to be the predominant mechanism that governs the sorption process^27,32,33^. When more NH_4_^+^ is present in the wastewater, more ions couple to the active –COO^−^ groups in the hydrogel, hence the proportional increase in total sorption. In theory, this trend is expected to manifest until the available –COO^−^ sites are completely saturated with ammonium ions^34^. Detailed equilibrium studies are warranted for a more accurate understanding of the sorption kinetics and mechanism and to predict further sorption behavior. In this study, we used industrially produced hydrogels without any physical and chemical modifications. As such, although the removal efficiencies achieved were 68% to 80%, depending on the ammonium concentration, improvements are needed to further increase the ammonium removal efficiencies. Furthermore, selective sorption of ammonium by hydrogels, in the presence of competing ions and organics, needs to be improved as seen in Table S1. To do so, the performance of the hydrogels in terms of removal efficiency and selectivity for ammonium can be improved through addition and alteration of functional groups to increase the affinity of the hydrogel for NH_4_^+^, and modification of surface porosity to increase sorption sites.

No significant changes in total sorption were observed within a pH range of 5 to 8, which means that the ammonium sorption process is not affected by any fluctuations in the wastewater pH. As observed in Supplementary Fig. S2b, the –COOH and –COO^−^ groups exhibit a buffering action that resists drastic pH change up to a certain extent^35,36^, which is known to be a typical behavior of PAA-based hydrogels. This finding suggests that, in a practical situation, PAA hydrogels can be used under a wide range of pH conditions, at least until the buffering capacity is reached.

In this study, regeneration was successfully achieved using mild acid washing at pH 4.0 with minimal loss of sorption performance. However, in practice the method of regeneration is largely dependent on the type of hydrogel selected and its material properties (e.g. stability to thermal degradation, robustness to physical compression), with typical approaches including physical compression, heating, and acid/base washing. The commercial hydrogel used in this proof of concept has a very high water uptake ratio because it is designed to be a superabsorbent polymer. As a result, the concentration of ammonium chloride in the regenerant stream is more dilute than desired for practical application. This issue can be addressed by tailoring the properties of hydrogels such as increasing crosslinking density which directly reduces the water uptake of hydrogels^37^, and/or altering the concentration and type of functional groups to control selectivity and strength of binding. In this fashion, we can potentially reduce the chemical requirements in regeneration, Hence, an increase in the end product concentration can be expected. This allows for practical ammonium recovery using available technologies for concentrated wastewater streams^14^. To further reduce the cost, natural sources such as starch can be used as a starting material for the fabrication of the hydrogels^38^ and other natural materials such as clays can be incorporated to produce hybrid hydrogels with reportedly improved mechanical and sorption properties and lower cost^39^. In addition to thorough materials development, another part of our future work is an assessment of the potential impacts of wastewater composition on long-term and multiple re-use. Overall, the findings are especially encouraging as, despite the fact that hydrogels were used without any physical and chemical modifications, ammonium sorption capacities and removal efficiencies were within the range of what can be achieved using various natural zeolites (with capacities in the range of 1.5–30.6 mg N/g)^18,40^.

Furthermore, the significance of the use of hydrogel sorbents lies in the versatility of this technology. Hydrogels can be placed in a conventional continuous stirred-tank reactor (CSTR), sequencing batch reactors (SBRs) or carrousels with wastewater effluent pumped continuously to the reactor at low residence times (i.e. <15 minutes). This allows for retrofitting of the concept into existing wastewater treatment plants. Equally important, it eliminates the need for a filter type configuration with periodic back-flushing which is necessary when operating with other sorbents, for example zeolites^41^. Finally, contrary to these filter type configurations, the regeneration process can be completely independent without affecting the mainstream process. Our findings therefore represent a first step towards mainstream ammonium recovery from domestic wastewater, thereby allowing wastewater utilities to transform their resource management from a linear to a circular and more sustainable approach.

## Materials and Methods

### Hydrogel preparation and characterization

A poly(acrylic acid)-based (PAA) hydrogel was selected on the basis of its functionality: PAA contains carboxylic acid groups that were proven to exhibit high binding affinity for NH_4_^+^ ions in aqueous solutions^26,27^. Furthermore, these functional groups are readily ionizable and responsive to pH^42^, which, in theory, would allow for mild hydrogel regeneration^43^.

2.5 g dry weight of PAA hydrogel (RST Solutions, Australia) was pre-washed by immersing in 1 L distilled water then 250 mL ethanol (70% v/v) for 3 days, with replacement of water and ethanol every 12 hours to remove any unreacted impurities. The hydrogels were then dehydrated using 100% (v/v) ethanol, filtered with a 100 mesh nylon screen and dried at 50 °C for 24 hours. ATR-FTIR spectroscopy (Nicolet iS5 FT-IR, Thermo Fisher, USA) was performed to confirm the presence of acrylic acid as the functional groups in the hydrogels (see Supplementary Fig. S1). Each spectrum was collected by accumulating 80 scans at a resolution of 4 cm^−1^ at room temperature.

### Wastewater characterization

Fresh unfiltered wastewater was collected from a local wet well in a residential zone in Brisbane only receiving household wastewater and immediately stored at 4 °C to minimize biological transformation of the sewage. Prior to the experimental runs, the wastewater was brought to ambient temperatures (20 ± 3 °C). Freshly collected wastewater was used for every set of experiments with characterization as shown in Table 1.

**Table 1:** Characterization of the domestic wastewater used in the experiments.

Parameter	Values
Ammonium (mg NH_4_–N L^−1^)^(a)^	39.3 ± 9.7
Nitrate (mg NO3–N L^−1^)	Negligible
Soluble COD (mg L^−1^)	171 ± 27
Phosphorus (mg PO_4_–P L^−1^)	6.6 ± 0.3
Temperature (°C)	20 ± 3
Calcium (mg L^−1^)	40.7 ± 2.8
Potassium (mg L^−1^)	24.6 ± 2.2
Magnesium (mg L^−1^)	25.2 ± 1.8
Sodium (mg L^−1^)	138 ± 12

^a^The value shown is the average NH4-N concentration of the sewage used in all experiments. In total, four batches of wastewater were used, one fresh batch for each set of experiment. Error estimates are standard deviation from all batches, measured in triplicates.

### Ammonium sorption testing

Three sets of 4-hour batch experimental runs were conducted. The first set of experiments (n = 3) was conducted to test the concept by determining the sorption performance of PAA hydrogels under normal sewage conditions. Experiments were carried out in flask reactors using 500 mL of raw domestic wastewater containing 2.5 g dry weight of hydrogels, which was equivalent to an ammonium loading of 7.1 ± 1.1 g NH_4_–N/kg hydrogel. All solutions were gently stirred at 100 rpm using a magnetic stirrer. To monitor the changes in NH_4_–N concentrations, 1 mL aliquots were collected from the settled liquid at t = 0, 5, 10, 30, 60 min and 4 hrs and immediately filtered with a 0.45 µm syringe filter.

The second set of experiments (n = 3) was conducted to investigate the effect of the NH_4_–N concentration on the overall sorption capacity of the hydrogels as well as on the achievable effluent NH_4_–N concentrations. From the initial average NH_4_-N concentration (~50 mg/L), the wastewater concentration was doubled to 90 mg/L and 180 mg/L to investigate the sorption performance of the hydrogels at higher concentrations. Ammonium chloride (ACS reagent, Sigma Aldrich USA) was added to increase the NH_4_–N of the wastewater to 90 mg/L and 180 mg/L respectively.

The third set of experiments (n = 3) was conducted to determine the impact of sewage pH on the sorption performance of the hydrogels. To do so, sorption tests were carried out at pH range of 5.0–8.0 at an influent concentration of 35 mg N/L and contact time of 60 minutes. The pH of the wastewater was adjusted by adding 1.0 mol/L HCl or NaOH. All sorption experiments followed the same conditions described above unless otherwise stated.

### Hydrogel regeneration and reusability

Further experiments were performed to determine the regeneration ability of the PAA hydrogels over multiple cycles. 2.5 g dry weight of hydrogel was immersed in 500 mL raw domestic wastewater and stirred at 100 rpm for 30 minutes. Afterwards, the NH_4_–N-loaded hydrogels were filtered using a fine nylon screen mesh. Regeneration was performed by mild acid washing (i.e. pH 4) of the retained hydrogels using hydrochloric acid as a desorbing agent and regenerant. The hydrogel was allowed to regenerate by slowly adding 1.0 M of HCl while being stirred continuously until the volume of the hydrogels collapsed and the pH reached 4.0. Samples were then collected from the spent liquid to determine the concentration of the recovered NH_4_–N. After regeneration, the hydrogels were filtered from the spent regenerant and then immersed in 500 mL of fresh domestic wastewater for the second sorption cycle. 1.0 M NaOH was used to neutralize any pH decrease at the beginning of the second sorption cycle caused by residual HCl in the hydrogels. The whole procedure was repeated five times to achieve a multicycle sorption-desorption process.

### Chemical analysis

COD concentrations were determined by means of COD cuvette tests (Merck, range 25–1500 mg/L). Elements (e.g. Na, Ca, K) were measured using inductively coupled plasma optical emission spectrometry (Perkin Elmer ICP-OES Optima 7300DV, Perkin Elmer, USA). Flow injection analysis (FIA) was used to determine the concentrations of NH_4_–N and PO_4_–P. The pH of the effluent during sorption experiments was monitored using a handheld pH probe (Thermo Fisher, USA).

### Data availability

All data generated or analyzed during this study are included in this published article (and its Supplementary Information files).

## Electronic supplementary material

Supplementary Information
