# Supplementary material for: Rapid removal of ammonium from domestic wastewater using polymer hydrogels
Source: Sci Rep. 2018 Feb 13;8:2912. doi: 10.1038/s41598-018-21204-4 (PMC5811486; doi:10.1038/s41598-018-21204-4)
Supplement: Supplementary file 1 — Supplementary Information [file 41598_2018_21204_MOESM1_ESM.doc]

Supporting Information

**Rapid removal of ammonium from domestic wastewater using polymer hydrogels**

**Heidy Cruz^a^, Paul Luckman^b^, Thomas Seviour**^c^**, Willy Verstraete^d^*,* Bronwyn Laycock^b^, and Ilje Pikaar^a,e*^**

^a^School of Civil Engineering, The University of Queensland, QLD 4072, Australia

^b^School of Chemical Engineering, The University of Queensland, QLD 4072, Australia

^c^Singapore Centre for Environmental Life Sciences Engineering, Nanyang Technological University, 637551, Singapore

^d^Center for Microbial Ecology and Technology (CMET), Ghent University, Coupure Links 653, 9000 Gent, Belgium

^e^Advanced Water Management Centre (AWMC), The University of Queensland, QLD 4072, Australia

*Correspondence should be addressed to: Ilje Pikaar, The School of Civil Engineering,

The University of Queensland, St. Lucia, QLD 4072, Australia

Phone: +61 7 3345 1389; E-mail: [i.pikaar@uq.edu.au](mailto:i.pikaar@uq.edu.au)

**Figure S1. ATR FTIR spectra of PAA hydrogels.** Each spectrum was collected by accumulating 80 scans at a resolution of 4 cm-1 recorded at room temperature. The spectra contains all the characteristic peaks of poly(acrylic acid).


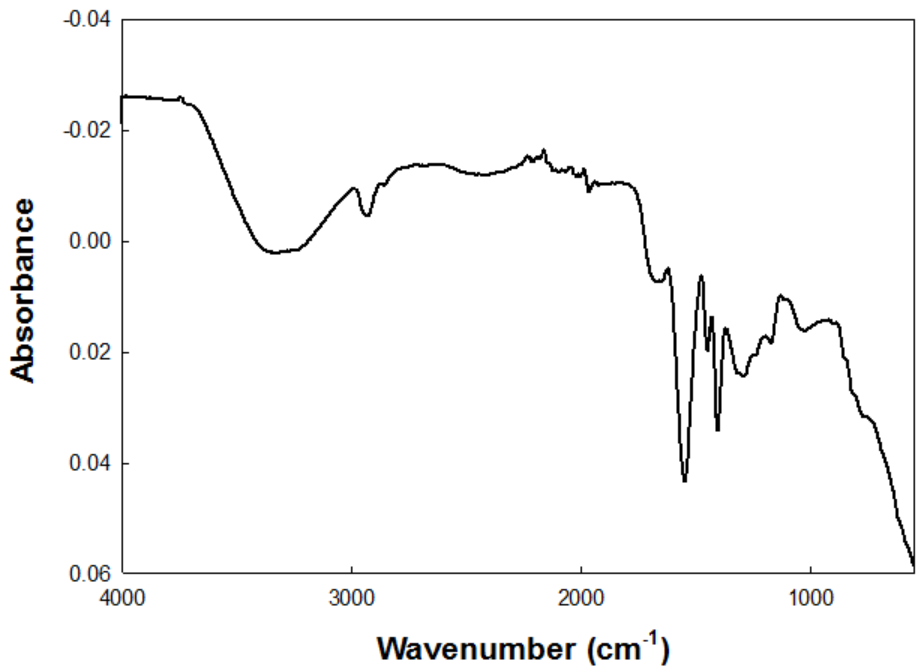


**Figure S2. (a) Adsorption capacity as a function of initial pH and (b) pH profile of wastewater effluent with varying initial pH during adsorption.** 1.0 mol/L HCl or NaOH was added to adjust the pH of the wastewater. Sorption conditions: C_0_ = 35 mg N/L, Contact time = 1h, T = 23 °C, natural pH = 7.1.


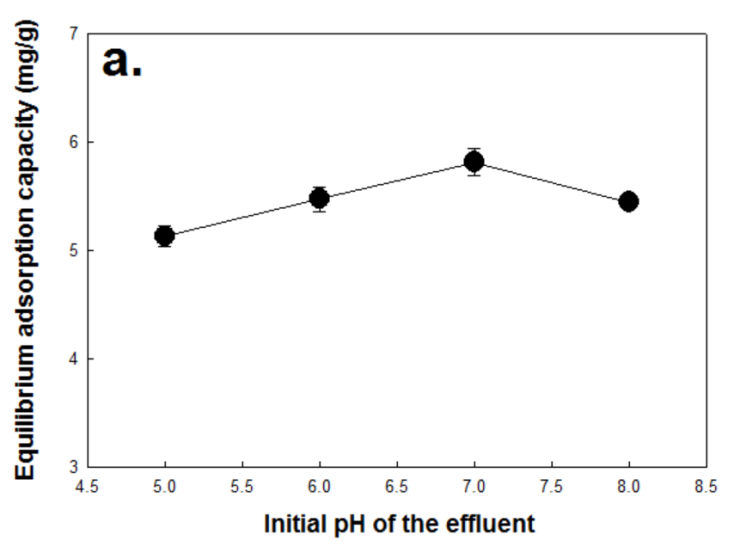

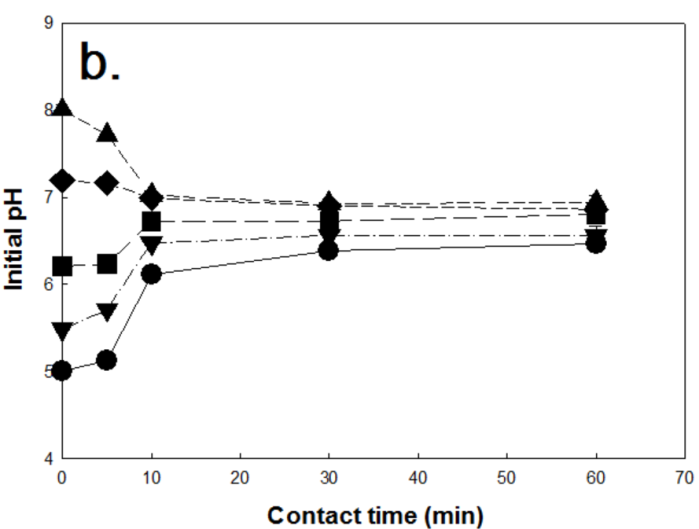


**Table S1.** Metals concentrations in domestic wastewater before and after adsorption

| **Analyte** | **Average Concentrations (mg/L)** | | | |
| --- | --- | --- | --- | --- |
|  | **t = 0** | **t = 4h  50 mg N/L** | **t = 4h  100 mg N/L** | **t = 4h  200 mg N/L** |
| Aluminum | 0.04 | 0.48 | 0.39 | 0.12 |
| Boron | 0.07 | 0.05 | 0.07 | 0.05 |
| Barium | 0.04 | 0.00 | 0.00 | 0.01 |
| Calcium | 40.73 | 2.60 | 2.49 | 5.42 |
| Copper | 0.10 | 0.14 | 0.17 | 0.25 |
| Iron | 0.25 | 0.16 | 0.18 | 0.15 |
| Potassium | 24.58 | 7.22 | 9.28 | 10.92 |
| Magnesium | 25.22 | 1.40 | 1.44 | 2.85 |
| Manganese | 0.05 | 0.00 | 0.00 | 0.01 |
| Sodium | 137.69 | 314.72 | 372.02 | 391.58 |
| Phosphorus | 10.91 | 9.69 | 10.79 | 8.66 |
| Sulfur | 25.28 | 25.46 | 27.18 | 20.84 |
| Zinc | 0.07 | 0.05 | 0.05 | 0.06 |
